# Mitochondrial genome of *Taiwania circumdata* (Coleoptera: Chrysomelidae: Cassidinae) and phylogenetic analysis

**DOI:** 10.1080/23802359.2017.1383205

**Published:** 2017-09-25

**Authors:** Xing-Zhuo Yang, Xiao-Peng Li, Chun-Li Wen, Cheng-Lin Jia, Li Zhang, Ming-Long Yuan

**Affiliations:** State Key Laboratory of Grassland Agro-Ecosystems, College of Pastoral Agricultural Science and Technology, Lanzhou University, Lanzhou, Gansu, People's Republic of China

**Keywords:** Leaf beetles, Cassidinae, mitochondrial DNA, evolution, phylogeny

## Abstract

In this study we sequenced and annotated the nearly complete mitochondrial genome (mitogenome) of *Taiwania circumdata* (Coleoptera: Chrysomelidae: Cassidinae), an important insect pest on sweetpotato and water spinach in Southern China. This mitogenome was 13,546 bp long and encoded 13 protein-coding genes (PCGs), 19 transfer RNA genes (tRNAs) and 2 ribosomal RNA unit genes. The *T. circumdata* mitogenome with an A + T content of 77.9% presented a positive AT-skew (0.126) and a negative GC-skew (−0.160). Eleven PCGs started with a typical ATN codon, whereas the remaining two PCGs used TTG (*nad1*) and AAT (*cox1*) as the initial codon. All the 19 tRNAs had a typical secondary cloverleaf structure, except for *trnS1* (AGN) which lacked the dihydrouridine arm. Phylogenetic analyses using Bayesian inference and maximum likelihood methods based on the concatenated nucleotide sequences of 13 PCGs recovered a phylogeny of Bruchinae+ ((Galerucinae + Chrysomelinae) + (Criocerinae + Cassidinae)). In Cassidinae, *T. circumdata* and *Laccoptera ruginosa* formed a clade, which was sister to three *Cassida* species.

## Introduction

Cassidinae is one of the most species-rich groups in Chrysomelidae, with approximately 6000 described species (Chaboo 2007). To better understand the diversity and phylogeny of Cassidinae, we sequenced and annotated the nearly complete mitochondrial genome (mitogenome) of *Taiwania circumdata*. This species is a serious insect pest on sweetpotato and water spinach in southern China. Adult specimens were collected from Nanchang City, Jiangxi Province, China, in August 2016. Samples have been deposited in College of Pastoral Agricultural Science and Technology, Lanzhou University, Lanzhou, China. The total genomic DNA was extracted from a single specimen using a DNeasy Tissue Kit (Qiagen). The *T. circumdata* mitogenome was amplified with a set of universal and specific primers, and sequenced in both directions.

We obtained the nearly complete mitogenome of *T. circumdata*, with 13,546 bp long (GenBank accession number MF946562). The region that we failed to sequence in *T. circumdata* was located between *rrnS* and *nad2*, and generally contained three transfer RNA genes (tRNAs; *trnI*, *trnQ* and *trnM*) and a putative control region. This mitogenome encoded 13 protein-coding genes (PCGs), 19 tRNAs, two ribosomal RNA unit genes (*rrnL* and *rrnS*). The order and orientation of the mitochondrial genes was identical to the inferred ancestral arrangement of insects (Boore 1999). Gene overlaps were found at six gene junctions and involved a total of 35 bp; the longest overlap (11 bp) existed between *trnE* and *trnF*. A total of 22 bp intergenic spacers were present in five positions, ranging in size from 1 to 17 bp.

The nucleotide composition of the *T. circumdata* mitogenome was biased toward A and T, with an A + T content of 77.9% on the J-strand. This mitogenome presented a positive AT-skew (0.126) and a negative GC-skew (−0.160), as found in most insect mitogenomes. The *rrnL* was 1265 bp long with an A + T content of 82.1%. Among the 13 PCGs, the lowest A + T content was 70.3% in *cox1*, while the highest was 85.6% in *atp8*. Eleven PCGs started with a typical ATN codon: five (*nad2*, *cox2*, *nad3*, *nad4L*, and *nad5*) with ATT and six (*atp8*, *atp6*, *cox3*, *nad4*, *nad6*, and *cob*) with ATG. The remaining two PCGs started with AAT (*cox1*) and TTG (*nad1*). Four PCGs terminated with TAA or TAG, whereas the remaining nine terminated with an incomplete stop codon TA or T which could be corrected via post-transcriptional polyadenylation. All of the 19 tRNAs had a typical cloverleaf secondary structure, except for *trnS1* (AGN) which lacked the dihydrouridine arm.

**Figure 1.**
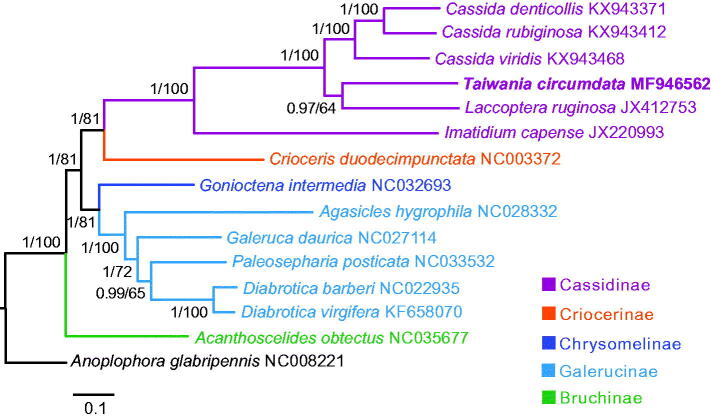
Mitochondrial phylogeny of 14 leaf beetles based on the concatenated nucleotide sequences of 13 mitochondrial protein-coding genes. Numbers on branches are Bayesian posterior probabilities (left) and Bootstrap values (right).

The concatenated nucleotide sequences of 13 PCGs in fourteen leaf beetles, representing five subfamilies, were used in phylogenetic analyses. *Anoplophora glabripennis* from the family Cerambycidae was used as an outgroup. Phylogenetic analyses were performed with Bayesian inference in MrBayes 3.2.3 (Ronquist et al. 2012) and maximum likelihood in RAxML 8.2.10 (Stamatakis 2014). We determined nucleotide substitution models for each PCG by jModelTest 2.1.7 (Darriba et al. 2012). Both analytical methods consistently recovered a phylogeny of Bruchinae + ((Galerucinae + Chrysomelinae) + (Criocerinae + Cassidinae)) (Figure 1). In Cassidinae, *T. circumdata* and *Laccoptera ruginosa* formed a clade, as sister to three *Cassida* species.
